# Lipid Indicators and Low Bone Mass Among Taiwanese Adults: A Cross-Sectional Association Study

**DOI:** 10.3390/nu18203349

**Published:** 2026-10-13

**Authors:** Hung-Shuo Ko, Chiao-Wei Chang, Chyi-Huey Bai

**Affiliations:** 1 Department of General Medicine, Chang Gung Memorial Hospital at Linkou, Taoyuan 333423, Taiwan; 2 School of Medicine, College of Medicine, Taipei Medical University, Taipei 110301, Taiwan; 3 School of Pharmacy, College of Pharmacy, Taipei Medical University, Taipei 110301, Taiwan; 4 Department of Public Health, School of Medicine, College of Medicine, Taipei Medical University, Taipei 110301, Taiwan; 5 School of Public Health, College of Public Health, Taipei Medical University, Taipei 110301, Taiwan

**Keywords:** bone mass, TC/HDL-C, lipid metabolism, bone homeostasis, adult population, NAHSIT

## Abstract

**Background & Aims:** This study investigated the associations of lipid indicators, particularly the total cholesterol to high-density lipoprotein cholesterol ratio (TC/HDL-C), with low bone mass in adults. **Methods:** This cross-sectional study included 6389 participants aged ≥19 years from the Nutrition and Health Survey in Taiwan (2013–2020). Two study-defined low-bone-mass phenotypes were evaluated as research phenotypes rather than densitometric diagnoses: central-site low bone mass, defined as a T-score < −1.0 at either the lumbar spine (L1–L4) or the lower of the bilateral femoral neck T-scores, and total-body low bone mass, defined as a total-body T-score < −1.0. Multivariable logistic regression models and restricted cubic spline analysis were performed to examine associations of lipid indicators with low bone mass. Subgroup analyses by sex, age, and age-defined menopausal status were exploratory. **Results:** Among 6389 participants, 1069 had total-body low bone mass. Total-body low bone mass showed more evident associations with lipid-related markers than central-site low bone mass. Each 10 mg/dL increase in HDL-C was associated with higher odds of total-body low bone mass (adjusted odds ratio [OR] = 1.080, 95% confidence interval [CI]: 1.018–1.147, *p* = 0.011). Although the linear association between TC/HDL-C and low bone mass was not statistically significant, spline analysis revealed a significant nonlinear association with total-body low bone mass, with a turning point at 4.39 (*p* for nonlinearity = 0.016). Exploratory analyses suggested effect modification by age and age-based grouping. **Conclusions:** Total-body low bone mass showed more evident associations with lipid indicators than central-site low bone mass. HDL-C was linearly associated with low bone mass, whereas TC/HDL-C showed an exploratory nonlinear association, supporting its potential role as a readily available metabolic correlate of skeletal status.

## 1. Background

Osteoporosis is a chronic systemic bone disease characterized by decreased bone mineral density (BMD) and deterioration of bone microarchitecture, leading to increased bone fragility and higher risk of fractures, particularly in older adults, thereby imposing a substantial financial burden on healthcare systems [1]. Low bone mass is widely recognized as an important precursor to osteoporosis, reflecting an early stage of skeletal deterioration before overt osteoporosis develops. Identifying factors associated with low bone mass is therefore essential for timely prevention and risk reduction. Since 1990, there has been a continuous increase in the global burden of disability-adjusted life years (DALYs) associated with osteoporosis [2], and the acceleration of population aging [3] worldwide underscores the growing importance of managing this disease. Aside from the well-established risk factors such as age, sex, and weight loss, recent research has focused on the role of metabolic disorders, particularly disorders of lipid metabolism, in the pathogenesis of osteoporosis.

Disorders of lipid metabolism have been increasingly implicated in skeletal health, as they share common pathogenic pathways and risk factors with low bone mass and osteoporosis [4]. Growing evidence suggests that lipid dysregulation may disturb bone homeostasis early in the disease process, even before osteoporosis becomes clinically apparent. Because lipid metabolism is involved in bone formation and remodeling, abnormalities in lipid profiles may contribute to reduced bone mass [5]. In particular, elevated cholesterol levels have been shown to inhibit osteoblast differentiation and proliferation while enhancing osteoclast activity, thereby promoting bone loss [6]. Beyond circulating lipids, metabolic alterations may affect bone homeostasis through adipose tissue dysfunction, chronic inflammation, and increased bone marrow adiposity, which may favor adipogenic over osteogenic differentiation of mesenchymal stem cells [7]. Collectively, these findings suggest that lipid metabolism may play an important role in the development of low bone mass.

Although lipid metabolism is closely linked to bone metabolism, the association between lipid profiles and bone mass remains uncertain. Previous studies evaluating conventional lipid parameters, including total cholesterol (TC), high-density lipoprotein cholesterol (HDL-C), low-density lipoprotein cholesterol (LDL-C), and triglyceride (TG), have yielded inconsistent results, with some reporting inverse associations, some null findings, and others positive associations with osteoporosis or related bone outcomes [8,9,10]. These inconsistencies suggest that the relationship between lipid metabolism and skeletal health is complex and may not be adequately captured by individual lipid measures alone. The TC/HDL-C ratio is an easily accessible composite lipid marker that reflects overall cardiometabolic risk [11] and has also been linked to insulin resistance, a condition potentially relevant to bone loss [12,13]. While a recent study suggested a U-shaped association between the TC/HDL-C ratio and osteoporosis in older women [14], evidence regarding its relationship with low bone mass remains limited.

Therefore, this study aimed to investigate the associations of lipid indicators, particularly the TC/HDL-C ratio, with central-site and total-body low bone mass among adults aged 19 years and older using data from the Nutrition and Health Survey in Taiwan (NAHSIT).

## 2. Materials and Methods

### 2.1. Study Design and Participants

This cross-sectional study pooled participant-level data from two NAHSIT survey waves (2013–2016 and 2017–2020), which aimed to monitor the health and nutrition status of the general population in Taiwan. NAHSIT employed a multistage, stratified, clustered, probability sampling design to recruit participants representing the civilian, non-institutionalized Taiwan population across all ages. The dataset comprised demographic characteristics, questionnaire responses, laboratory measurements, and medical health status. The sampling design proceeded through successive strata of seven major geographic regions, counties/cities, districts and neighborhoods, and households, with all age groups sampled within each cluster. Since 2013, each NAHSIT survey year has yielded samples representative of Taiwan nationally and at the county/city level.

**Figure 1:**
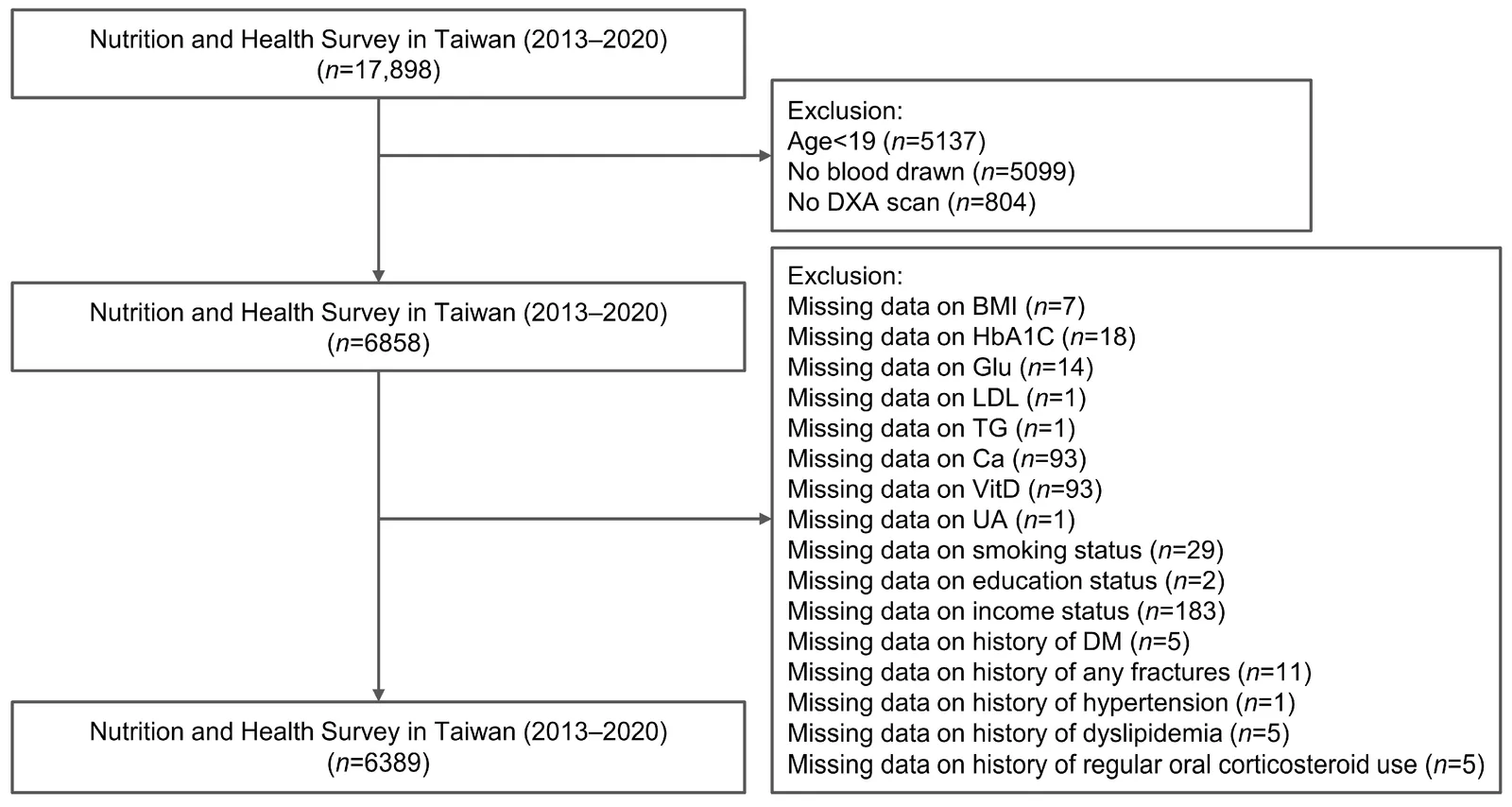
Flow chart of participant selection in NAHSIT (2013–2020). The right-pointing arrows indicate excluded participants and the corresponding reasons for exclusion. Abbreviations: BMI, body mass index; HbA1c, Glycated hemoglobin; Glu, Fasting plasma glucose; LDL-C, Low-density lipoprotein cholesterol; TG, Triglyceride; Ca, Calcium; VitD, 25-hydroxyvitamin D; UA, Uric acid; DM, diabetes.

Data collection comprised two stages: a household visit including face-to-face interview and dietary assessment, followed by venipuncture and physical examinations including DXEA. Participation in the second stage was incomplete, and the analytic sample is therefore smaller than the first-stage sample (Figure 1). Identical definitions and inclusion criteria were applied across waves, and blood collection procedures, lipid assays, and DXEA protocols and equipment were identical, performed in the same mobile examination vehicle.

Sampling weights, stratum, and cluster identifiers wer not included in the approved dataset for this secondary analysis and were therefore not incorporated. Because participants within the same neighborhood and household are likely to be positively correlated with respect to both lipid profile and bone density, the model-based standard errors, 95% confidence intervals, and statistical tests reported here do not account for within-cluster correlation, and are likely to be underestimated and the corresponding *p*-values anti-conservative. The estimates are intended to evaluate associations within the analytic sample rather than to provide nationally representative estimates.

Due to legal restrictions imposed by the government of Taiwan under the “Personal Information Protection Act”, data are available only through a formal proposal to the Health and Welfare Data Science Center (HWDC), Ministry of Health and Welfare, Taiwan. This secondary analysis was approved by the Institutional Review Board of Taipei Medical University (N202508071, approval date: 1 September 2025).

In total, 17,898 participants were included. The exclusion criteria were as follows: (1) age younger than 19 years (*n* = 5137), (2) no blood drawn (*n* = 5099), (3) no dual-energy X-ray absorptiometry (DXA) assessments (*n* = 804), (4) missing data on covariates including body mass index (BMI, *n* = 7), glycated hemoglobin (HbA1c, *n* = 18), fasting plasma glucose (Glu, *n* = 14), LDL-C (*n* = 1), TG (*n* = 1), calcium (Ca, *n* = 93), 25-hydroxyvitamin D (VitD, *n* = 93), uric acid (UA, *n* = 1), smoking status (*n* = 29), educational level (*n* = 2), income (*n* = 183), diabetes history (*n* = 5), history of any fracture (*n* = 11), hypertension history (*n* = 1), dyslipidemia history (*n* = 5), regular use of oral corticosteroids (*n* = 5). Finally, 6389 participants were included in the current study (Figure 1). The primary analyses used a complete-case approach, with participants missing data for required covariates excluded sequentially according to the flowchart shown in Figure 1.

### 2.2. Definition of Low Bone Mass

BMD was measured using a mobile dual-energy X-ray absorptiometry (DXA) device (Lunar Prodigy, GE Healthcare, Madison, WI, USA), operating in basic analysis mode. All scans were performed by trained radiologic technicians. T-scores were derived from the manufacturer’s reference populations (v113): the USA (Combined NHANES ages 20–30 years/Lunar ages 20–40 years) reference population for the total body and AP spine regions, and NHANES III–derived reference values for the femoral neck. Consistent with the International Society for Clinical Densitometry (ISCD) nomenclature, T-scores were based on sex-matched young adult reference data, preserving age trends and relevant characteristics to align with the study objectives.

Two study-defined, T-score-based low-bone-mass phenotypes were evaluated. Both were treated as operationally defined research indicators rather than densitometric diagnoses, and WHO diagnostic categories were not applied to either. Central-site low bone mass was defined as a T-score < −1.0 at either the lumbar spine (L1–L4) or the lower of the bilateral femoral neck T-scores. Although these are skeletal sites recognized by the ISCD for diagnostic classification [15], the T-score threshold in the present study was used only to define a research phenotype across the full cohort aged ≥19 years and was not intended to apply WHO diagnostic categories to all participants, particularly younger adults. Total-body low bone mass was defined as a total body BMD T-score < −1.0. The total body is not an ISCD-recognized diagnostic site; this variable was therefore used as an operationally defined research indicator of overall skeletal mineral status rather than as a densitometric diagnosis, and WHO diagnostic categories were not applied to it.

### 2.3. Lipid Measurement

All serum lipid traits were measured according to standard protocols. TC was measured by the CHOD-PAP enzymatic colorimetric method [16]. HDL-C and LDL-C were measured using homogeneous enzymatic colorimetric assays [17,18], and TG was measured using the GPO-PAP enzymatic colorimetric method [19]. The TC/HDL-C ratio was calculated by dividing TC by HDL-C.

### 2.4. Definitions of Covariates

The following covariates were included: age, sex, BMI, HbA1c, serum Glu, HDL-C, LDL-C, TC, TG, creatinine levels, estimated glomerular filtration rate (eGFR), UA, Ca, and VitD. BMI was calculated as weight divided by height squared (kg/m^2^). The eGFR was calculated using the four-variable Modification of Diet in Renal Disease (MDRD-4) equation [20]. Smoking status was determined by the questionnaire, “Have you ever smoked a total of 100 cigarettes or more during your lifetime?” Those who answered “yes” were categorized as ever smokers. Education level was divided into three levels according to the years of formal education: <6 years, 6–12 years, and >12 years. Income level was also classified into three levels based on average monthly household income: <30,000 New Taiwan dollars (TWD), 30,000–< 50,000 TWD, and ≥50,000 TWD. Diabetes mellitus (DM), history of fracture at any site, hypertension, dyslipidemia, and history of corticosteroid use were categorized based on self-reported physician diagnoses or participant-provided information.

### 2.5. Statistical Analysis

Baseline characteristics were compared according to total-body low-bone-mass status. Continuous variables were presented as mean ± standard deviation (SD) and compared using the unpaired *t* test. Categorical variables were presented as counts (percentages) and compared using the Pearson chi-square test or Fisher’s exact test, as appropriate. Differences between respondents and non-respondents (age and sex), and between the two survey waves (age, sex, lipid concentrations, total body T-scores and central site T score), were quantified using standardized mean differences (SMD), with an SMD below 0.10 considered to indicate negligible imbalance.

Separate models were fitted for total-body and central-site low bone mass. For each outcome, each lipid indicator (HDL-C, TC, LDL-C, TG, and the TC/HDL-C ratio) was entered separately as the exposure variable. Univariable and multivariable logistic regression analyses were performed to estimate odds ratios (ORs) and 95% confidence intervals (CIs) for the associations of lipid indicators with low bone mass phenotypes.

Restricted cubic spline models were used to assess potential nonlinear associations. Candidate models with three, four, and five knots were compared using the Akaike information criterion (AIC). The knots were placed at the 10th, 50th, and 90th percentiles in the three-knot model; at the 5th, 35th, 65th, and 95th percentiles in the four-knot model; and at the 5th, 27.5th, 50th, 72.5th, and 95th percentiles in the five-knot model. The three-knot model yielded the lowest AIC and was therefore selected for the final analyses. For the spline plots, model predictions and their 95% confidence limits were transformed to the probability scale using the inverse-logit function. The turning point was assessed by spline analysis. Nonlinearity was tested using the Wald chi-square test. The turning points were defined as the exposure values corresponding to the minimum of the fitted spline curve over the prediction grid, and the resulting values were subsequently used as fixed breakpoints in exploratory two-piece logistic regression analyses.

Four regression models were constructed. Model 1 was unadjusted. Model 2 was adjusted for age, sex, and BMI. Model 3 was further adjusted for HbA1c, serum glucose, triglyceride, calcium, vitamin D, uric acid, estimated glomerular filtration rate, smoking status, education level, monthly income, and histories of diabetes mellitus, fracture, hypertension, dyslipidemia, and chronic corticosteroid use. Model 4 included all covariates in Model 3 except triglycerides to assess whether adjustment for this correlated lipid measure influenced the estimated associations. Multicollinearity was assessed separately for each fully adjusted model using variance inflation factors (VIFs), with a VIF ≥ 5 considered indicative of potentially important multicollinearity.

Stratified analyses were performed by age, sex, and age-defined menopausal status because sex- and age-specific patterns are a primary focus of this analysis. Since actual menopausal status was unavailable, women aged >55 years were classified as postmenopausal using an age-based proxy, according to a recent cohort study in Taiwan [21]. Analyses were stratified by sex and by age-defined menopausal status among women. These stratifications did not alter the underlying study-defined T-score criterion. An age threshold of 65 years was used for age-stratified analyses. Exploratory logistic regression models assessed interactions of continuous TC/HDL-C with sex, age group, and age-defined menopausal status, adjusting for Model 3 covariates with model-specific age adjustment. Continuous age was retained in the sex-interaction model, represented by age group in age-group models, and omitted from the menopausal-status model because of collinearity with the age-based indicator. Two-way and three-way interactions were tested using likelihood ratio tests comparing nested models with and without the relevant interaction term, retaining all lower-order terms and using identical observations. The same specifications were applied in outlier sensitivity analyses. Two sets of sensitivity analyses were conducted to assess the robustness of the observed associations, and an additional Z-score-based analysis was attempted. First, analyses were repeated in the following age- and sex-defined subsets: (a) participants aged >25 years; (b) male and female participants aged >25 years, analyzed separately; (c) men aged ≥50 years; and (d) women aged ≥45 years. The exclusion of participants aged ≤25 years was intended to assess the potential influence of ongoing bone mineral accrual in younger adults, given longitudinal evidence that much of peak bone mass is attained by the mid-twenties, with variation by sex and skeletal site [21,22]. The male age threshold followed ISCD recommendation favoring T-scores in men aged ≥50 years [23]. The female age threshold was based on the World Health Organization (WHO) description of natural menopause typically occurring between ages 45 and 55 years [24], and it was not used to establish menopausal status. Second, analyses were repeated after excluding outliers of TC/HDL-C, defined as values below Q1 − 3 × IQR or above Q3 + 3 × IQR, where Q1 and Q3 denote the first and third quartiles, respectively, and IQR denotes the interquartile range. Third, we attempted a Z-score-based sensitivity analysis in younger adults (men aged <50 years and women aged <45 years), defining below the expected range for age as a Z-score ≤ −2.0 at either the lumbar spine (L1–L4) or femoral neck. However, the number of Z-score-defined events was age-dependent and limited (61 of 1005 men and 19 of 903 women), resulting in model instability and preventing reliable estimation of the fully adjusted multivariable models. Therefore, inferential results from this analysis were not reported.

Because the sampling design variables required for design-based variance estimation were unavailable, design-adjusted confidence intervals could not be computed. As a quantitative sensitivity assessment, we instead derived a critical design effect (DEFF_crit) for each association. The design effect (DEFF) is the ratio of the variance of an estimate under a complex sampling design to its variance under simple random sampling of the same size [25]. DEFF_crit is defined as the value of DEFF at which the 95% confidence interval of the corresponding odds ratio would include the null (OR = 1.0). DEFF_crit was computed on the log-odds scale as (z/1.96)^2^, where z = ln(OR)/SE and SE = [ln(upper 95% CI) − ln(lower 95% CI)]/3.92. Larger values indicate greater robustness to unmeasured within-cluster correlation; associations not reaching conventional significance under model-based variance estimation (DEFF_crit < 1) are reported as NS. No value of the design effect was assumed.

All analyses were performed using R version 4.5.0, and a two-sided *p* value < 0.05 was considered statistically significant.

## 3. Results

### 3.1. Baseline Characteristics

After applying inclusion and exclusion criteria, 6389 participants were included in the study. Differences between respondents (*n* = 6389) and non-respondents (*n* = 6372) were negligible in magnitude for both age and sex (SMD 0.08 and 0.02, respectively). Differences between the two survey waves were small, with SMDs of 0.16 for age, 0.02 for sex, 0.17 for HDL-C, 0.15 for TC, 0.07 for TG, 0.04 for LDL-C, 0.05 for TC/HDL ratio, 0.13 for total-body T-scores and 0.06 for central-site T-scores.

Participants were divided into two groups based on total-body T-score: 5320 participants had T-scores ≥ −1.0 and 1069 participants met the study-defined total-body T-score-based low-bone-mass phenotype (T-score < −1.0), as shown in Table 1. Participants with study-defined low-bone-mass phenotype were significantly older and more likely to be female. They also had lower BMI, monthly average income, and educational attainment. Additionally, they had a higher prevalence of diabetes, hypertension, and a history of fractures. A greater proportion of non-smokers was observed in the low-bone-mass group compared to participants with T-scores ≥ −1.0. Laboratory findings showed participants with low bone mass had higher HbA1c and fasting plasma glucose levels, and lower TG, calcium, creatinine, and eGFR. Additionally, although both TC and HDL-C levels were higher, the TC/HDL-C ratio was lower compared to those with T-scores ≥ −1.0.

### 3.2. The Associations Between Continuous Lipids and Low Bone Mass Phenotypes in Logistic Regression

Table 2 presents the associations of continuous lipid profiles (HDL-C, TC, LDL-C, and TG) and TC/HDL-C with two low-bone-mass phenotypes, based on both univariable and multivariable logistic regression models. All VIFs in these fully adjusted lipid-association models were <5, indicating no evidence of potentially important multicollinearity. For clinical interpretability, ORs for HDL-C, TC, LDL-C, and TG were reported per 10 mg/dL increase. In the unadjusted models, higher HDL-C and TC levels were positively associated with both total-body and central-site low bone mass, whereas the TC/HDL-C ratio was inversely associated with both outcomes. These associations were estimated on the logit scale and are expressed as odds ratios. However, after adjustment for the covariates in Model 3, only HDL-C remained significantly associated with both low-bone-mass phenotypes. Notably, each 10 mg/dL increase in HDL-C was associated with 8.0% higher odds of total-body low bone mass (adjusted OR = 1.080, 95% CI: 1.018–1.147, *p* = 0.011) and 6.3% higher odds of central-site low bone mass (adjusted OR = 1.063, 95% CI: 1.014–1.114, *p* = 0.012). In contrast, no significant associations were observed for TC, LDL-C, and the TC/HDL-C ratio after multivariable adjustment in Model 3. TG was also not significantly associated with either outcome in Model 4. The estimates obtained from Model 4, which excluded TG from the covariate set, were generally similar to those from Model 3. Overall, these findings suggest that total-body low bone mass showed more evident associations with lipid metabolism than central-site low bone mass.

Sensitivity analysis (*n* = 6373) was also conducted by excluding 16 participants with extreme TC/HDL-C values based on the ±3 IQR criterion, showing a generally similar direction to those in the main analysis (Table S1). Critical design effects for these associations are presented in Supplementary Table S3. The unadjusted associations of HDL-C and the TC/HDL-C ratio were highly robust (DEFF_crit 31.23 and 13.33 for the total-body phenotype; 9.98 and 4.42 for the central-site phenotype). However, associations in Table 2 and Table S1 that remained significant after full adjustment had DEFF_crit values between 1.0 and 1.8, indicating that modest within-cluster correlation could render them non-significant.

### 3.3. Nonlinear Associations Between Lipids and Low Bone Mass

**Figure 2:**
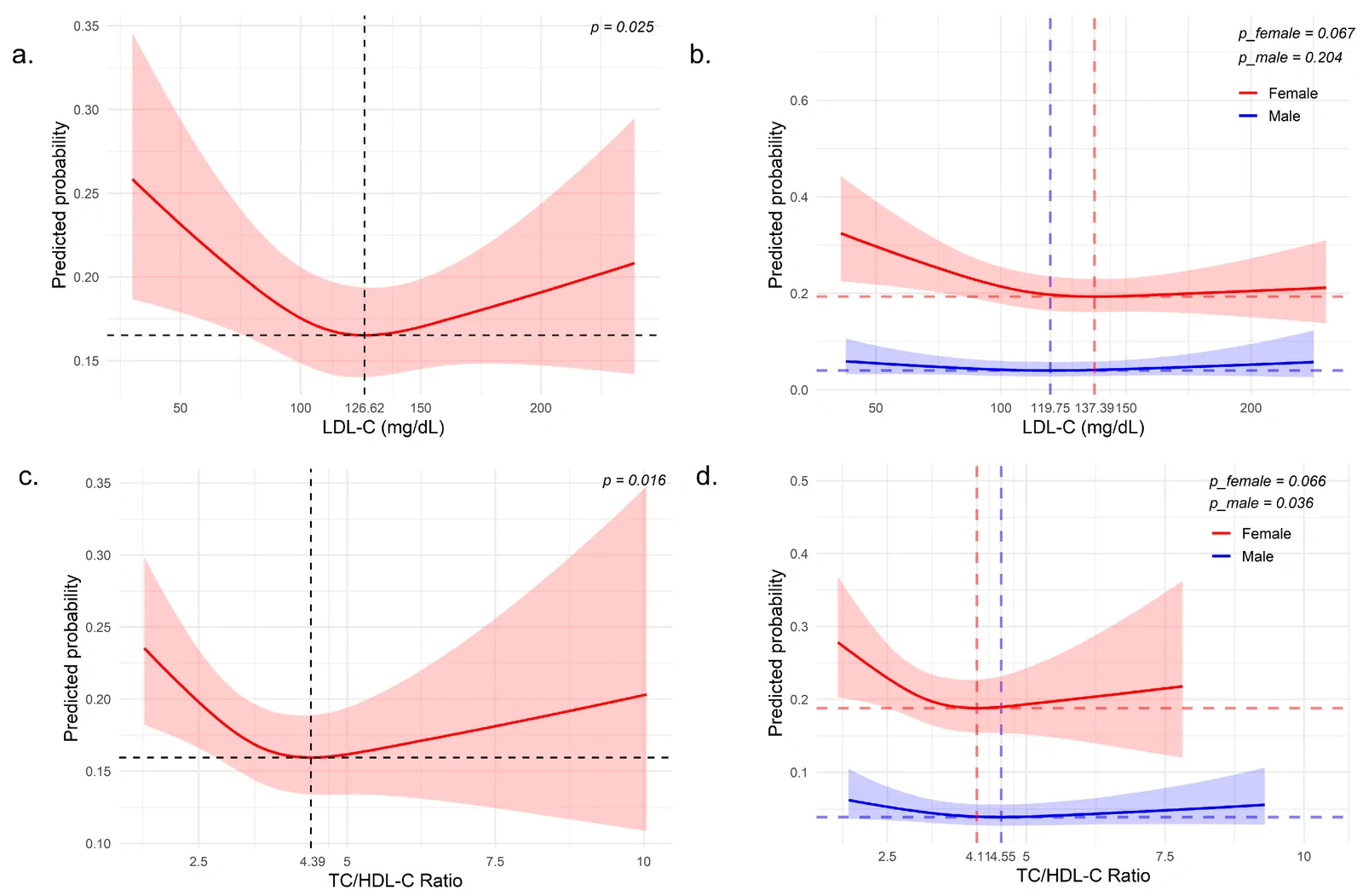
Restricted cubic spline analyses of lipid traits and total-body low bone mass. (**a**) Spline analysis of LDL-C in all participants. (**b**) Spline analysis of LDL-C in male (blue) and female (red) participants. (**c**) Spline analysis of the TC/HDL-C ratio in all participants. (**d**) Spline analysis of the TC/HDL-C ratio in male (blue, *n* = 3163, low bone mass = 244) and female (red, *n* = 3226, low bone mass = 825) participants. Vertical dashed lines indicate the estimated turning points. Solid curves represent model-predicted probabilities, and shaded areas indicate pointwise 95% confidence intervals. Adjusted for all covariates in model 3 (see Methods).

We further assessed the nonlinear relationships between continuous lipid profiles and predicted probabilities of study-defined low-bone-mass using restricted cubic spline analysis, applying the same covariate adjustment strategy as that used in the multivariable logistic regression models. Figure 2 illustrated the dose–response relationships of LDL-C and the TC/HDL-C ratio with study-defined total-body low-bone-mass phenotype, based on predicted probabilities derived from the fully adjusted models. After controlling for all covariates, the TC/HDL-C ratio showed a significant nonlinear association with total-body low bone mass, with a turning point around 4.39 (*p* for nonlinearity = 0.016; Figure 2c). In addition, LDL-C showed a modest but statistically significant nonlinear association with total-body low bone mass, with a turning point around 126.62 (*p* for nonlinearity = 0.0248; Figure 2a). By contrast, TG, HDL-C, and TC (*p* for nonlinearity = 0.057) did not exhibit significant nonlinear patterns and therefore were not presented in the figure. For the central-site phenotype the overall spline patterns were generally similar, but the associations were weaker and less distinct than those observed for the total-body phenotype. The turning point from predicted probability is imprecisely estimated and should not be interpreted as a clinically actionable cut-point.

Exploratory two-piece logistic regression was performed using the data-derived TC/HDL-C turning point of 4.39. Below this threshold, a higher TC/HDL-C ratio was significantly associated with lower odds of low bone mass in both the unadjusted model (OR: 0.754, 95% CI: 0.672–0.845, *p* < 0.01; Table 3) and the fully adjusted model (adjusted OR: 0.818, 95% CI: 0.698–0.959, *p* = 0.013; Table 3), corresponding to a 18.2% reduction in odds per one-unit increase. Above this threshold, each one-unit increase in the TC/HDL-C ratio was associated with a 14.0% increase in the odds of low bone mass (adjusted OR: 1.140, 95% CI: 1.016–1.278, *p* = 0.025; Table 3).

A similar exploratory threshold analysis was conducted for LDL-C, with a turning point at 126.62. Below this threshold, LDL-C was significantly associated with lower odds of total-body low bone mass in the fully adjusted model (adjusted OR: 0.993, 95% CI: 0.988–0.998; *p* = 0.010; Table 3). No significant association was observed across models above the threshold.

In exploratory sex-stratified analyses, a significant nonlinear association between the TC/HDL-C ratio and the study-defined total-body low-bone-mass phenotype was observed in males, with a turning point at 4.55 (*p* for nonlinearity = 0.0358; Figure 2d). By contrast, no significant pattern was detected in females. However, above the threshold of 4.11, the TC/HDL-C ratio was positively associated with the total-body phenotype in the fully adjusted Model 3 (adjusted OR: 1.355, 95% CI: 1.030–1.783, *p* = 0.030; Table 3). For LDL-C, no significant nonlinear association was identified in either sex in the spline analyses (Figure 2b). Nevertheless, in females, below the threshold (137.39 mg/dL), higher LDL-C levels were associated with significantly lower odds of total-body phenotype (adjusted OR: 0.991; 95% CI: 0.986–0.997; *p* = 0.002; Model 3; Table 3). In males, above the data-derived threshold of 119.75 mg/dL, LDL-C was inversely associated with the outcome in Model 3 (adjusted OR: 0.995, 95% CI: 0.991–1.000, *p* = 0.049; Table 3).

Sensitivity analyses were conducted in age- and sex-defined subsets, including participants aged >25 years, both overall and separately by sex, men aged ≥50 years (68.2%), and women aged ≥45 years (72%). The shapes of the TC/HDL-C association curves and the results of the nonlinearity tests were broadly consistent with the main analyses (Figure S1). Among participants aged >25 years, evidence of nonlinearity remained significant overall (*p* = 0.020) and in men (*p* = 0.032), but not in women (*p* = 0.103). Similarly, nonlinearity was significant in men aged ≥50 years (*p* = 0.012), but not in women aged ≥45 years (*p* = 0.074).

After excluding outliers of TC/HDL-C (±3 IQR), the estimated turning points for LDL-C and TC/HDL-C changed slightly from 126.62 to 126.71 and from 4.39 to 4.52, respectively. The OR directions and general shape of the association were similar to those in the main analyses; however, the statistical evidence for nonlinearity was attenuated. For TC/HDL-C, the nonlinearity test changed to *p* = 0.058, and above the revised turning point of 4.52 the adjusted association was no longer statistically significant (OR: 1.222, 95% CI: 0.948–1.576, *p* = 0.122; Figure S2c and Table S2). Although the point estimate above the threshold increased relative to the main analysis, its confidence interval widened. These results indicate uncertainty in the data-derived turning point and provide only partial support for the nonlinear finding. Critical design effects for the threshold analyses (Table S3) exceeded 6 only for the unadjusted TC/HDL-C association below the turning point; segments above the turning point were the lowest (1.3–1.9) in the all-participant analysis, consistent with the attenuation observed after exclusion of extreme observations, which also shifted the turning points themselves. These analyses are therefore regarded as exploratory.

### 3.4. Interaction Effect Analyses

**Figure 3:**
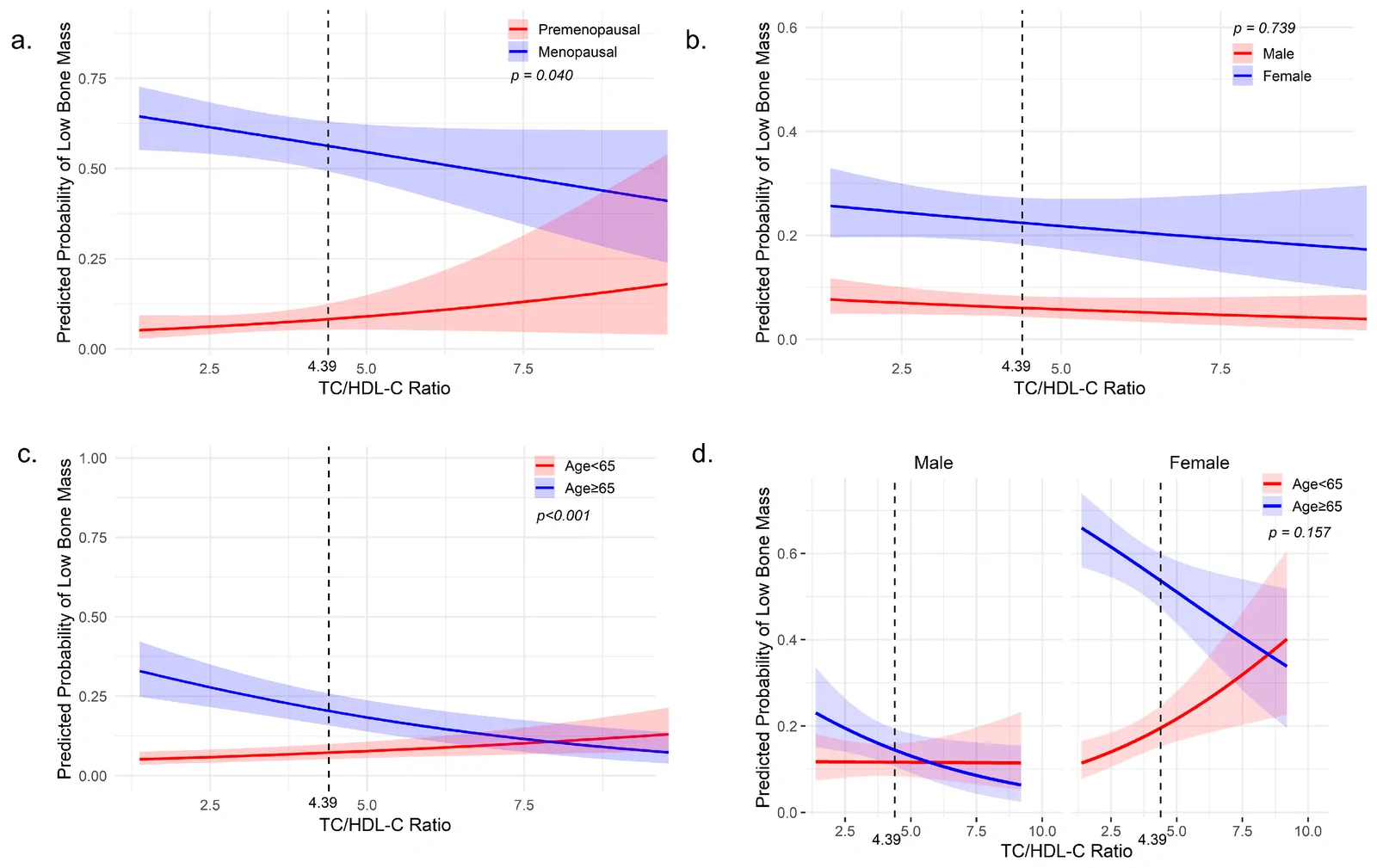
Exploratory interaction analyses of TC/HDL-C ratio with age-defined menopausal status, sex, and age in relation to study-defined total-body T-score-based low-bone-mass phenotype. (**a**) Age-defined menopausal status (red: premenopausal, *n* = 1468, events = 65; blue: menopausal, *n* = 1758, events = 760). (**b**) Sex (red: male, *n* = 3163, events = 244; blue: female, *n* = 3226, events = 825). (**c**) Age (red: age < 65 years, *n* = 3861, events = 339; blue: age ≥ 65 years, *n* = 2528, events = 730). (**d**) Predictions by age group and sex from the three-way interaction model (red: age < 65 years; blue: age ≥ 65 years and sample sizes: male < 65 years, *n* = 1840, events = 102; male ≥ 65 years, *n* = 1323, events = 142; female < 65 years, *n* = 2021, events = 237; female ≥ 65 years, *n* = 1205, events = 588). The vertical dashed line at TC/HDL-C = 4.39 marks the turning point from the overall primary spline analysis for reference. Solid curves represent model-predicted probabilities, and shaded areas indicate pointwise 95% confidence intervals. The *p* value in panel D is for the overall three-way interaction. Covariate adjustment and prediction settings are described in Section 2.5. Panel B adjusts for continuous age; panels A, C, and D do not additionally include continuous age. Panel A is restricted to women.

We conducted exploratory interaction analyses to examine whether the association between the TC/HDL-C ratio and total-body low bone mass varied by age, sex, and age-defined menopausal status. As shown in Figure 3, significant effect modification was observed for age-defined menopausal status among women and age (*p* for interaction 0.040 and <0.001, respectively; Figure 3a,c). The predicted probability of low bone mass increased with increasing TC/HDL-C ratio among women aged ≤55 years (Figure 3a) and participants aged <65 years (Figure 3c), whereas the opposite pattern was observed among age-defined postmenopausal women and those aged ≥65 years. Neither the interaction between the TC/HDL-C ratio and sex (*p* = 0.739; Figure 3b) nor the three-way interaction among the TC/HDL-C ratio, age group, and sex (*p* = 0.157; Figure 3d) was statistically significant in the main analysis.

After exclusion of 16 extreme TC/HDL-C observations, the interaction between the TC/HDL-C ratio and sex remained nonsignificant (*p* = 0.377; Figure S3B), whereas the three-way interaction among the TC/HDL-C ratio, age group, and sex changed from nonsignificant to significant (*p* = 0.041; Figure S3D). Because this change occurred after removal of a very small number of observations, the three-way interaction finding appears sensitive to extreme values and should be interpreted cautiously.

## 4. Discussion

### 4.1. Overall Findings

This study examined the associations of multiple lipid indicators, including HDL-C, TC, LDL-C, TG, and the TC/HDL-C ratio, with low bone mass among Taiwanese adults and yielded several findings. First, total-body low bone mass showed more evident associations with lipid-related markers than central-site low bone mass. Second, among the indicators examined, HDL-C showed the most consistent linear association after multivariable adjustment, although the magnitude was modest; the TC/HDL-C ratio and LDL-C showed nonlinear patterns in the spline analyses. Third, exploratory analyses suggested that the association between the TC/HDL-C ratio and low bone mass varied by age and by age-based menopausal classification, with more pronounced associations among participants aged <65 years and women aged ≤55 years. These findings differed in robustness: the unadjusted associations of HDL-C and the TC/HDL-C ratio were the most stable, whereas the fully adjusted and threshold-based findings were sensitive to model specification and should be interpreted as exploratory.

### 4.2. Total-Body and Central-Site Low Bone Mass in Relation to Lipid Indicators

Total-body low bone mass and central-site low bone mass reflect different aspects of skeletal health. Total-body low bone mass may better represent overall skeletal mineral deficiency. In contrast, central-site low bone mass has greater clinical relevance because it is based on standard diagnostic skeletal sites for osteoporosis and fracture risk assessment. In our study, total-body low bone mass showed more evident associations with lipid indicators than central-site low bone mass. Differences in the skeletal regions assessed may contribute to these findings, but the present analysis cannot establish their explanation. This interpretation is consistent with prior evidence showing positive correlations of skeletal muscle mass, skeletal muscle index, and fat-free mass with BMD [26]. However, no formal diagnostic-performance comparison was performed, and the cross-sectional design cannot establish that the total-body phenotype is more sensitive or represents an earlier temporal stage of skeletal deterioration.

### 4.3. Lipid Indicators in Relation to Low Bone Mass

The mechanisms linking lipid-related indices to bone health remain unclear. Shared cardiovascular risk factors and pathophysiologic pathways between osteoporosis and atherosclerosis may partly explain these associations [27]. Previous studies have reported U-shaped or nonlinear associations between HDL-C and bone outcomes [28,29]. Importantly, the present study further suggests that not only HDL-C, but also total cholesterol and different cholesterol-related subtypes, may be associated with skeletal metabolism. LDL-C and the TC/HDL-C ratio demonstrated prominent nonlinear patterns, suggesting that different lipid indicators may reflect distinct aspects of lipid-related skeletal alterations. These associations may reflect broader metabolic alterations beyond the direct effects of circulating lipids. Although BMI was adjusted for in our analyses, it may not fully capture the metabolic effects of adipose tissue. Adipose tissue dysfunction and inflammation may alter the bone marrow microenvironment and favor adipogenic over osteogenic differentiation of mesenchymal stem cells, potentially affecting bone remodeling [7]. However, these mechanisms cannot be directly assessed in the present cross-sectional study.

In our study, HDL-C was associated with greater odds of low bone mass in Model 3. Although clinical studies have reported inconsistent findings regarding the relationship between HDL-C and osteoporosis in adults, several studies have reported associations consistent with our findings. Two cross-sectional studies conducted in the general adult population in China and the United States of America (USA) respectively reported inverse associations between HDL-C and BMD [26,27,30,31]. Moreover, a post hoc analysis of the Aspirin in Reducing Events in the Elderly (ASPREE) clinical trial revealed that higher HDL-C concentrations were linked to an increased fracture risk in healthy older adults [32]. Recently, a large prospective cohort study in the United Kingdom (UK) following 414,302 participants for a median period of 13.8 years demonstrated that elevated HDL-C levels were associated with a higher risk of fractures and osteoporosis [33]. Two meta-analyses also supported this finding [34,35]. However, these epidemiological associations should not be interpreted as evidence that HDL-C itself is harmful to bone and therefore warrant cautious interpretation and further mechanistic and prospective investigation.

In contrast, the association between TC, LDL-C, TG, and osteoporosis remains inconclusive. Most of the existing evidence comes from cross-sectional studies, limiting the ability to establish a definitive causal relationship [36,37,38,39]. Building on this uncertainty, recent studies have begun to examine the TC/HDL-C ratio as a potentially more informative lipid-related marker for bone health. A cross-sectional study of men in South Korea and another focusing on older women reported a negative relationship between the TC/HDL-C ratio and BMD [14,37]. However, a case–control study involving postmenopausal women with or without coronary artery disease found a positive correlation between the TC/HDL-C ratio and BMD [40]. These discrepancies may be attributed to different study designs, population characteristics, or the presence of systemic disease, underscoring the need for further research. Future studies should further clarify the associations between the TC/HDL-C ratio and osteoporosis.

In the exploratory analysis, we also observed a significant nonlinear association between the TC/HDL-C ratio and total-body low bone mass among adults, with both low and high ratios significantly associated with greater odds of low bone mass. The result was consistent with a previous study using data from the National Health and Nutrition Examination Survey (NHANES) in older adults [14] and suggested a similar pattern in a broader adult Taiwanese population. However, the present threshold should not be regarded as a validated cutoff because it was derived and tested in the same dataset. Moreover, statistical evidence for nonlinearity was attenuated after exclusion of extreme observations. This nonlinear pattern may reflect the complex interplay between lipid metabolism and bone physiology. A low TC/HDL-C ratio may result from lower total cholesterol or higher HDL-C, both of which may influence bone homeostasis through cholesterol-dependent cellular and hormonal pathways [41,42,43]. Conversely, a high TC/HDL-C ratio may reflect higher TC or lower HDL-C, which have been associated with increased bone turnover and oxidative stress, potentially affecting osteoblast–osteoclast balance and bone remodeling [44,45,46,47]. Exploratory interaction analyses suggested that the association between TC/HDL-C and low bone mass varied by age and age-defined menopausal status, with more pronounced associations among younger participants and women aged ≤55 years and classified as premenopausal proxy. Previous studies similarly reported that higher TC levels or TC/HDL-C ratios in healthy women aged <55 years were associated with lower femoral neck BMD or spine BMD [48,49]. In contrast, the association appeared attenuated in older participants or women classified as postmenopausal, potentially reflecting the dominant effects of estrogen decline and age-related skeletal changes [14,50,51]. Overall, TC/HDL-C may represent an easily obtainable metabolic correlate of skeletal status; however, these findings remain exploratory and require prospective confirmation.

### 4.4. Limitations

There were several limitations in our study. First, since these findings were derived from the cross-sectional design, causal inference between the lipids indicators and low bone mass risk cannot be established. Second, no Taiwan- or Asia-specific reference population was available within the software; T-scores were therefore derived from a US young adult reference. However, the analyses of association were therefore conducted within sex strata. Importantly, all participants were scanned on a single device using a single software version, analysis mode, and reference population, minimizing measurement heterogeneity within the study. Third, we applied T-score-based criteria across the adult age range. These variables were study-defined research phenotypes rather than densitometric diagnoses, and diagnostic labels were not applied to any participants. T-scores were retained because the study focused on an absolute, threshold-defined skeletal phenotype. Age-matched Z-scores would answer a different question by referencing participants to peers of the same age. Sensitivity analyses restricted to men aged ≥50 years, women aged ≥45 years, or excluding subjects ≤ 25 years yielded materially similar patterns (showed in Figure S1). We additionally attempted a Z-score-based sensitivity analysis in men aged <50 years and women aged <45 years. However, only 61 of 1005 men and 19 of 903 women were applied the ISCD criterion of Z ≤ −2.0; the age-dependent and limited number of events resulted in a model with non-convergence that precluded reliable estimation of the fully adjusted models. Fourth, the menopausal status in our study was determined based on age rather than actual clinical history, which may have introduced misclassification bias and overlap conceptually with the age-stratified analyses. Fifth, the complete-case approach and substantial reduction from the initial survey sample may have introduced selection bias. Sixth, residual confounding may remain because information on physical activity, dietary patterns, alcohol consumption, specific lipid-lowering therapies, hormone replacement or other sex-hormone-related treatments, osteoporosis medications, and other bone-active medications was unavailable or not included. Seventh, multiple lipid indicators, outcomes, nonlinear models, thresholds, and subgroup analyses were examined, increasing the possibility of type I error. Finally, the two NAHSIT waves were pooled for individual-level analyses without survey weights, strata, or clustering identifiers, as these variables are classified as highly sensitive and were not included in the approved dataset. Although the waves were comparable in composition (all SMD ≤ 0.17) and applied identical measurement protocols, period effects cannot be excluded. Because participants within the same neighborhood and household are likely to be positively correlated with respect to both lipid profile and bone density, the reported standard errors are likely underestimated and the *p*-values anti-conservative. To quantify this, we derived a critical design effect (DEFF_crit) for each association (Table S3): unadjusted HDL-C and TC/HDL-C associations were robust (4.2–31.2) in Table 2 and Table S1, whereas those remaining significant after full adjustment ranged from 1.0 to 1.8 and are presented as provisional. The findings describe associations within the pooled analytic sample rather than nationally representative estimates. Future longitudinal studies with more comprehensive covariate assessment and appropriate survey-design information are warranted to confirm these findings.

## 5. Conclusions

In conclusion, this cross-sectional study found that lipid-related markers were associated with study-defined low bone mass, with total-body low bone mass showing more evident associations than central-site low bone mass. HDL-C showed the most consistent linear association, although the magnitude was modest and sensitive to model specification. The TC/HDL-C ratio showed a nonlinear pattern in exploratory analyses. Exploratory analyses further suggested effect modification by age and by age-based grouping among women, with more pronounced associations among participants aged <65 years and women aged ≤55 years. Given the cross-sectional design and the provisional nature of the fully adjusted estimates, these findings should be regarded as hypothesis-generating and warrant confirmation in studies incorporating the full survey design.

## Figures and Tables

**Table 1:** Baseline characteristics according to the study defined total-body T-score-based low-bone-mass phenotype.

Characteristic	Total (*n* = 6389)	T-Score ≥ −1 (*n* = 5320)	T-Score < −1 (*n* = 1069)	*p*
Age, years	55.73 ± 17.15 ^a^	53.47 ± 17.09	67.00 ± 12.34 *	<0.001
Sex (*n*, %)				
Male	3163 (49.51) ^b^	2919 (54.87)	244 (22.83) *	<0.001
Female	3226 (50.49)	2401 (45.13)	825 (77.17) *	
BMI, kg/m^2^	24.74 ± 4.10	25.04 ± 4.16	23.26 ± 3.42 *	<0.001
HbA1c, %	5.86 ± 0.93	5.84 ± 0.92	5.96 ± 0.98 *	<0.001
Glu, mg/dL	105.93 ± 28.48	105.50 ± 28.08	108.08 ± 30.30 *	0.010
TC, mg/dL	184.59 ± 36.18	183.98 ± 35.87	187.63 ± 37.58 *	<0.001
HDL-C, mg/dL	51.17 ± 15.11	50.22 ± 14.63	55.89 ± 16.56 *	<0.001
LDL-C, mg/dL	116.20 ± 33.51	116.34 ± 33.28	115.49 ± 34.67	0.462
TC/HDL-C	3.80 ± 1.30	3.85 ± 1.31	3.55 ± 1.23 *	<0.001
TG, mg/dL	124.97 ± 86.78	125.94 ± 87.99	120.13 ± 80.33 *	0.034
Ca, mg/dL	9.32 ± 0.36	9.33 ± 0.36	9.30 ± 0.40 *	0.033
VitD, ng/mL	30.76 ± 10.98	30.82 ± 11.03	30.42 ± 10.71	0.259
Creatinine, mg/dL	0.82 ± 0.36	0.83 ± 0.36	0.77 ± 0.35 *	<0.001
eGFR, mL/min/1.73 m^2^	100.37 ± 29.74	101.00 ± 29.27	97.21 ± 31.81 *	<0.001
UA, mg/dL	5.71 ± 1.53	5.79 ± 1.53	5.30 ± 1.42 *	<0.001
Smoking (*n*, %)				
No	4519 (70.73)	3617 (67.99)	902 (84.38) *	<0.001
Yes	1870 (29.27)	1703 (32.01)	167 (15.62) *	
Education (*n*, %)				
<6 years	542 (8.48)	319 (6.00)	223 (20.86) *	<0.001
6~12 years	2073 (32.45)	1596 (30.00)	477 (44.62) *	
>12 years	3774 (59.07)	3405 (64.00)	369 (34.52) *	
Income (*n*, %)				
<30,000	4252 (66.55)	3377 (63.48)	875 (81.85) *	<0.001
30,000 ~ <50,000	1206 (18.88)	1107 (20.81)	99 (9.26) *	
≥50,000	931 (14.57)	836 (15.71)	95 (8.89) *	
Diabetes history (*n*, %)				
No	5619 (87.95)	4703 (88.40)	916 (85.69) *	0.014
Yes	770 (12.05)	617 (11.60)	153 (14.31) *	
Fracture history (*n*, %)				
No	4872 (76.26)	4115 (77.35)	757 (70.81) *	<0.001
Yes	1517 (23.74)	1205 (22.65)	312 (29.19) *	
Hypertension history (*n*, %)				
No	4536 (71.00)	3864 (72.63)	672 (62.86) *	<0.001
Yes	1853 (29.00)	1456 (27.37)	397 (37.14) *	
Dyslipidemia history (*n*, %)				
No	5605 (87.73)	4683 (88.03)	922 (86.25)	0.118
Yes	784 (12.27)	637 (11.97)	147 (13.75)	
Regular use of oral corticosteroids, %				
No	6376 (99.80)	5312 (99.85)	1064 (99.53)	0.052
Yes	13 (0.20)	8 (0.15)	5 (0.47)	

^a^ Continuous variables are presented as mean ± standard deviation. ^b^ Categorical variables are presented as number (percentage). * Statistically significant difference between groups (*p* < 0.05). Abbreviations: BMI, body mass index; HbA1c, Glycated hemoglobin; Glu, Fasting plasma glucose; TC, Total cholesterol; HDL-C, High-density lipoprotein cholesterol; LDL-C, Low-density lipoprotein cholesterol; TC/HDL-C, Total cholesterol to high-density lipoprotein cholesterol ratio; TG, Triglyceride; Ca, Calcium; VitD, 25-hydroxyvitamin D; UA, Uric acid; eGFR, Estimated glomerular filtration rate.

**Table 2:** Association between lipid profiles and study-defined T-score-based low-bone-mass phenotypes in adults using logistic regression.

	Total-Body Low Bone Mass (T-Score < −1.0)						Central-Site Low Bone Mass (Any T-Score < −1.0)					
Variables, Unit	Model 1 ^a^ OR (95% CI)	*p*	Model 3 ^b^ OR (95% CI)	*p*	Model 4 ^c^ OR (95% CI)	*p*	Model 1 ^a^ OR (95% CI)	*p*	Model 3 ^b^ OR (95% CI)	*p*	Model 4 ^c^ OR (95% CI)	*p*
HDL-C, per 10 mg/dL	1.264 (1.212, 1.318) *	<0.001	1.080 (1.018, 1.147) *	0.011	1.053 (0.996, 1.113)	0.068	1.111 (1.074, 1.148) *	<0.001	1.063 (1.014, 1.114) *	0.012	1.050 (1.005, 1.097) *	0.029
TC, per 10 mg/dL	1.028 (1.010, 1.047) *	0.003	1.003 (0.981, 1.026)	0.782	1.007 (0.984, 1.029)	0.568	1.014 (1.000, 1.028) *	0.049	1.013 (0.996, 1.030)	0.148	1.013 (0.996, 1.030)	0.130
TG, per 10 mg/dL	0.991 (0.983, 1.000) *	0.046	-	-	1.007 (0.997, 1.017)	0.160	0.998 (0.992, 1.003)	0.413	-	-	1.002 (0.995, 1.009)	0.652
LDL-C, per 10 mg/dL	0.992 (0.973, 1.012)	0.450	0.984 (0.961, 1.008)	0.195	0.985 (0.961, 1.008)	0.203	0.999 (0.985, 1.014)	0.911	1.003 (0.986, 1.021)	0.703	1.003 (0.986, 1.021)	0.706
TC/HDL-C	0.802 (0.755, 0.852) *	<0.001	0.933 (0.850, 1.024)	0.147	0.989 (0.919, 1.064)	0.764	0.920 (0.884, 0.957) *	<0.001	0.967 (0.910, 1.028)	0.289	0.985 (0.938, 1.034)	0.540

* Statistically significant association (*p* < 0.05). ^a^ Unadjusted. ^b^ Adjusted for age, sex, BMI, HbA1c, Glu, TG, UA, Ca, VitD, eGFR, smoking status, education level, monthly average income level, DM, fracture history, hypertension, dyslipidemia, regular use of corticosteroids. ^c^ Adjusted for covariates included in Model 3 except TG. Total-body low bone mass was defined as a total-body BMD T-score < −1.0. Central-site low bone mass was defined as a T-score < −1.0 based on the lower of the right or left femoral neck T-scores or the mean lumbar spine (L1–L4) T-score. Abbreviations: OR, odds ratio; CI, confidence interval; *p*, *p*-value; -: not available; BMI, body mass index; HbA1c, Glycated hemoglobin; Glu, Fasting plasma glucose; TC, Total cholesterol; HDL-C, High-density lipoprotein cholesterol; TC/HDL-C, Total cholesterol to high-density lipoprotein cholesterol ratio; TG, Triglyceride; Ca, Calcium; VitD, 25-hydroxyvitamin D; UA, Uric acid; eGFR, Estimated glomerular filtration rate; DM, diabetes.

**Table 3:** Exploratory threshold effect analyses of the TC/HDL-C ratio and per-unit LDL-C in relation to the study-defined total-body-T-score-based low-bone-mass phenotype.

Population with Inflection Point	Model 1 ^a^ OR (95% CI)	*p*	Model 2 ^b^ OR (95% CI)	*p*	Model 3 ^c^ OR (95% CI)	*p*
All participants						
TC/HDL-C ≤ 4.39	0.754 (0.672, 0.845) *	<0.001	0.905 (0.788, 1.039)	0.156	0.818 (0.698, 0.959) *	0.013
TC/HDL-C > 4.39	1.030 (0.932, 1.138)	0.568	1.143 (1.036, 1.261) *	0.008	1.140 (1.016, 1.278) *	0.025
LDL-C ≤ 126.62	0.995 (0.991, 0.999) *	0.007	0.995 (0.991, 1.000)	0.057	0.993 (0.988, 0.998) *	0.010
LDL-C > 126.62	1.003 (0.999, 1.008)	0.167	1.002 (0.996, 1.007)	0.584	1.002 (0.997, 1.008)	0.420
Male participants						
TC/HDL-C ≤ 4.55	0.981 (0.896, 1.073)	0.670	0.937 (0.836, 1.050)	0.264	0.898 (0.772, 1.045)	0.165
TC/HDL-C > 4.55	0.971 (0.858, 1.098)	0.636	1.027 (0.883, 1.193)	0.732	0.987 (0.798, 1.221)	0.905
LDL-C ≤ 119.75	1.002 (0.999, 1.005)	0.253	1.000 (0.997, 1.004)	0.888	0.998 (0.994, 1.002)	0.393
LDL-C > 119.75	0.998 (0.995, 1.002)	0.371	0.996 (0.992, 1.000)	0.055	0.995 (0.991, 1.000) *	0.049
Female participants						
TC/HDL-C ≤ 4.11	1.042 (0.891, 1.217)	0.607	0.922 (0.759, 1.119)	0.412	0.828 (0.661, 1.037)	0.101
TC/HDL-C > 4.11	1.114 (0.947, 1.309)	0.193	1.280 (1.057, 1.551) *	0.012	1.355 (1.030, 1.783) *	0.030
LDL-C ≤ 137.39	0.995 (0.991, 0.999) *	0.018	0.995 (0.989, 1.000) *	0.040	0.991 (0.986, 0.997) *	0.002
LDL-C > 137.39	1.000 (0.993, 1.007)	0.991	0.997 (0.989, 1.005)	0.488	0.997 (0.988, 1.006)	0.468

* Statistically significant association (*p* < 0.05). ^a^ Unadjusted. ^b^ Adjusted for age, sex, and BMI. ^c^ Adjusted for age, sex, BMI, HbA1c, Glu, TG, UA, Ca, VitD, eGFR, smoking status, education level, monthly average income level, DM, fracture history, hypertension, dyslipidemia, regular use of corticosteroids. Abbreviations: OR, odds ratio; CI, confidence interval; *p*, *p*-value; BMI, body mass index; HbA1c, Glycated hemoglobin; Glu, Fasting plasma glucose; TC, Total cholesterol; HDL-C, High-density lipoprotein cholesterol; TC/HDL-C, Total cholesterol to high-density lipoprotein cholesterol ratio; TG, Triglyceride; Ca, Calcium; VitD, 25-hydroxyvitamin D; UA, Uric acid; eGFR, Estimated glomerular filtration rate; DM, diabetes.

## Data Availability

Permission to access and analyze to the dataset was granted following a formal application process to the Health and Welfare Data Science Center (HWDC), Taiwan. The Institutional Review Board of Taipei Medical University approved this secondary analysis (N202508071, approval date: 1 September 2025). This study used anonymized secondary data, and no identifiable personal information is included. All analyses must be conducted within the HWDC under its data access and monitoring regulations, and only approved analytical outputs including tables and figures may be released from the Center.

## Supplementary Materials

The following supporting information can be downloaded at: https://www.mdpi.com/article/10.3390/nu18203349/s1. Figure S1. Restricted cubic spline analyses of the TC/HDL-C ratio in relation to the study-defined total-body T-score-based low-bone-mass phenotype in age- and sex-defined sensitivity subsets. (a) Spline analysis of the TC/HDL-C ratio in all participants age >25 (*n* = 6007, events = 1055). (b) Spline analysis of the TC/HDL-C ratio in male (blue, *n* = 2972, events = 239) and female (red, *n* = 3035, events = 816) participants age > 25. (c) Spline analysis of the TC/HDL-C ratio in male participants age ≥ 50 years (blue, *n* = 2158, events = 205). (d) Spline analysis of the TC/HDL-C ratio in female participants age ≥ 45 years (red, *n*=2323, events = 799). Vertical dashed lines indicate the estimated turning points. Solid curves represent model-predicted probabilities, and shaded areas indicate pointwise 95% confidence intervals. Adjusted for all covariates in model 3 (see Methods). Figure S2. Restricted cubic spline analyses of lipid traits in relation to the study-defined total-body T-score-based low-bone-mass phenotype after exclusion of extreme TC/HDL-C observations. (a) Spline analysis of LDL-C in all participants. (b) Spline analysis of LDL-C in male (blue) and female (Red). (c) Spline analysis of the TC/HDL-C ratio in all participants. (d) Spline analysis of the TC/HDL-C ratio in male (blue) and female (red) participants. Vertical dashed lines indicate the estimated turning points. Solid curves represent model-predicted probabilities, and shaded areas indicate pointwise 95% confidence intervals. Adjusted for all covariates in model 3 (see Methods). Figure S3. Exploratory interaction analyses of TC/HDL-C ratio with age-defined menopausal status, sex, and age in relation to study-defined total-body T-score-based low-bone-mass phenotype after exclusion of extreme TC/HDL-C observations. (a) Age-defined menopausal status (red: premenopausal; blue: menopausal). (b) Sex (red: male; blue: female). (c) Age (red: age < 65 years; blue: age ≥ 65 years). (d) Predictions by age group and sex from the three-way interaction model (red: age < 65 years; blue: age ≥ 65 years). The vertical dashed line at TC/HDL-C = 4.52 marks the turning point from the overall outlier sensitivity spline analysis for reference. Solid curves represent model-predicted probabilities, and shaded areas indicate pointwise 95% confidence intervals. The *p* value in panel D is for the overall three-way interaction. Covariate adjustment and prediction settings are described in Section 2.5. Panel B adjusts for continuous age; panels A, C, and D do not additionally include continuous age. Panel A is restricted to women. Table S1. Association between lipid profiles and study-defined T-score-based low-bone-mass phenotypes in adults using logistic regression. Table S2. Exploratory threshold effect analyses of the TC/HDL-C ratio and per-unit LDL-C in relation to the study-defined total-body T-score-based low-bone-mass phenotype after exclusion of extreme TC/HDL-C observations. Table S3. Critical design effects (DEFF_crit) for all reported associations between lipid indicators and the study-defined T-score-based low-bone-mass phenotypes.
