# Supplementary material for: Lipid Indicators and Low Bone Mass Among Taiwanese Adults: A Cross-Sectional Association Study
Source: Nutrients. 2026 Oct 13;18(20):3349. doi: 10.3390/nu18203349 (PMC13708668; doi:10.3390/nu18203349)
Supplement: Supplementary file 1 [file nutrients-18-03349-s001.zip › nutrients-4556910-supplementary.pdf]

Supplementary information

Lipid Indicators and Low Bone Mass Among Taiwanese Adults: A Cross-Sectional Association Study

Journal: Nutrients

Authors:

Hung-Shuo Ko[1,2], Chiao-Wei Chang[3], Chyi-Huey Bai[4, 5*]

[1]Department of General Medicine, Chang Gung Memorial Hospital at Linkou, Taoyuan 333423, Taiwan;

[2]School of Medicine, College of Medicine, Taipei Medical University, Taipei 110301, Taiwan

[3]School of Pharmacy, College of Pharmacy, Taipei Medical University, Taipei 110301, Taiwan

[4]Department of Public Health, School of Medicine, College of Medicine, Taipei Medical University, Taipei 110301, Taiwan

[5]School of Public Health, College of Public Health, Taipei Medical University, Taipei, Taiwan

***Corresponding Author**

Chyi-Huey Bai, PhD

School of Public Health, College of Public Health, Taipei Medical University, Taipei 110301, Taiwan

Telephone: +886-2-62002589 ext. 16013. E-mail: baich@tmu.edu.tw

Figure Legends

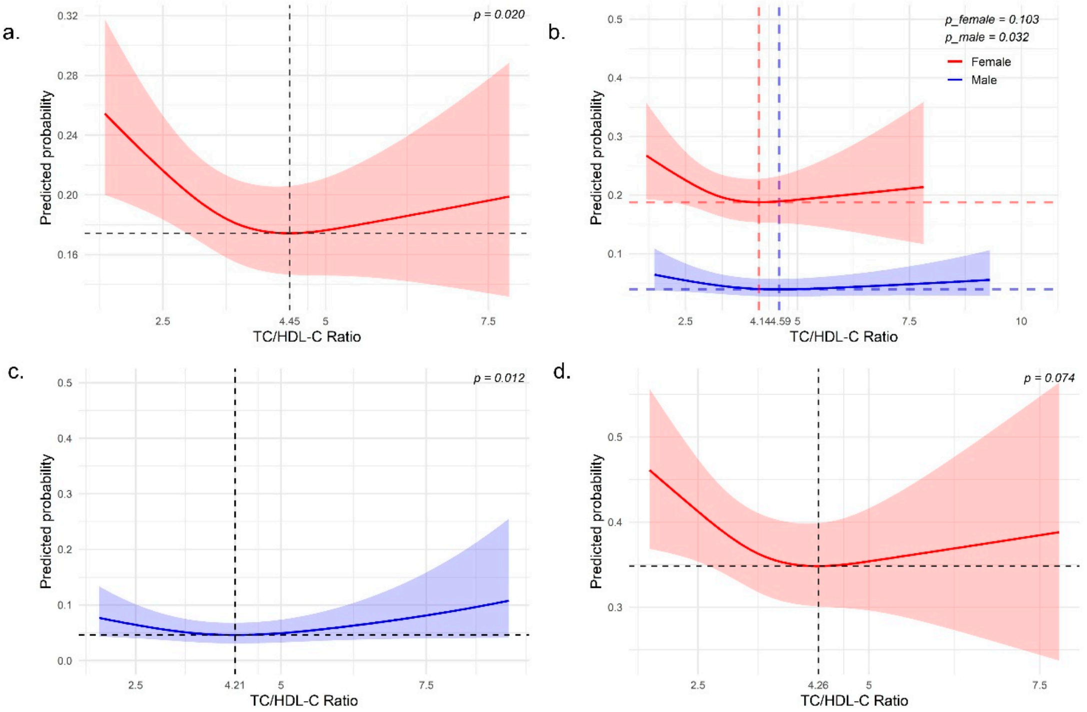

Figure S1. Restricted cubic spline analyses of the TC/HDL-C ratio in relation to the study-defined total-body T-score-based low-bone-mass phenotype in age- and sex-defined sensitivity subsets. (a) Spline analysis of the TC/HDL-C ratio in all participants age >25 (n = 6007, events = 1055). (b) Spline analysis of the TC/HDL-C ratio in male (blue, n = 2972, events = 239) and female (red, n = 3035, events = 816) participants age > 25. (c) Spline analysis of the TC/HDL-C ratio in male participants age ≥ 50 years (blue, n = 2158, events = 205). (d) Spline analysis of the TC/HDL-C ratio in female participants age ≥ 45 years (red, n=2323, events = 799). Vertical dashed lines indicate the estimated turning points. Solid curves represent model-predicted probabilities, and shaded areas indicate pointwise 95% confidence intervals. Adjusted for all covariates in model 3 (see Methods).

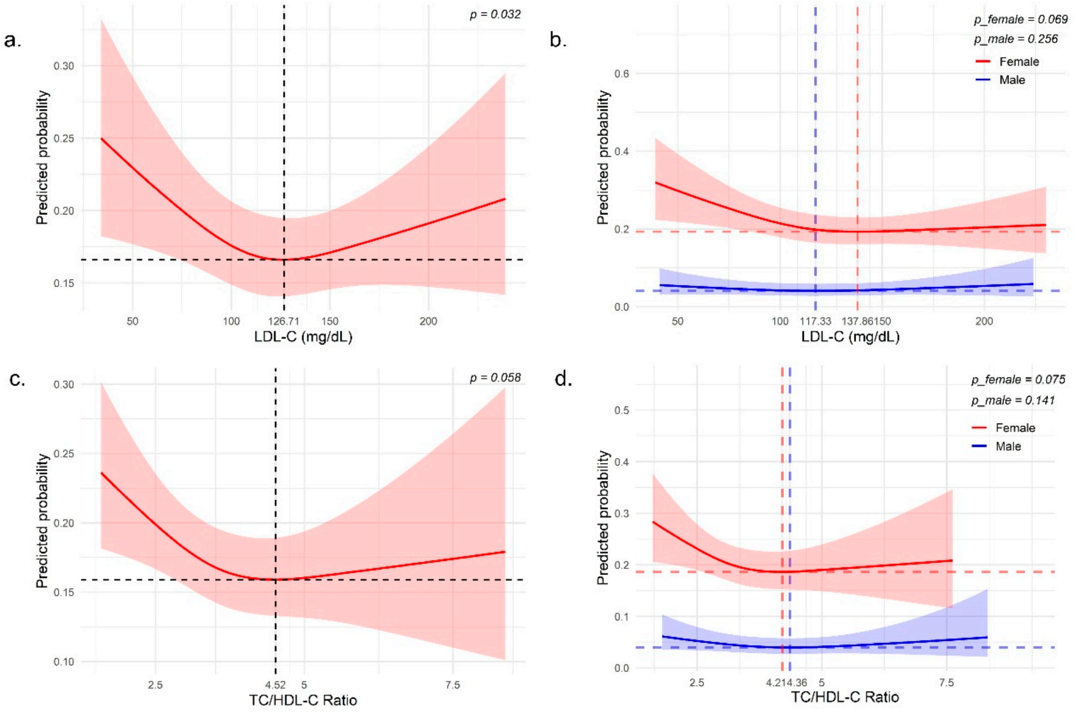

Figure S2. Restricted cubic spline analyses of lipid traits in relation to the study-defined total-body T-score-based low-bone-mass phenotype after exclusion of extreme TC/HDL-C observations. (a) Spline analysis of LDL-C in all participants. (b) Spline analysis of LDL-C in male (blue) and female (Red). (c) Spline analysis of the TC/HDL-C ratio in all participants. (d) Spline analysis of the TC/HDL-C ratio in male (blue) and female (red) participants. Vertical dashed lines indicate the estimated turning points. Solid curves represent model-predicted probabilities, and shaded areas indicate pointwise 95% confidence intervals. Adjusted for all covariates in model 3 (see Methods).

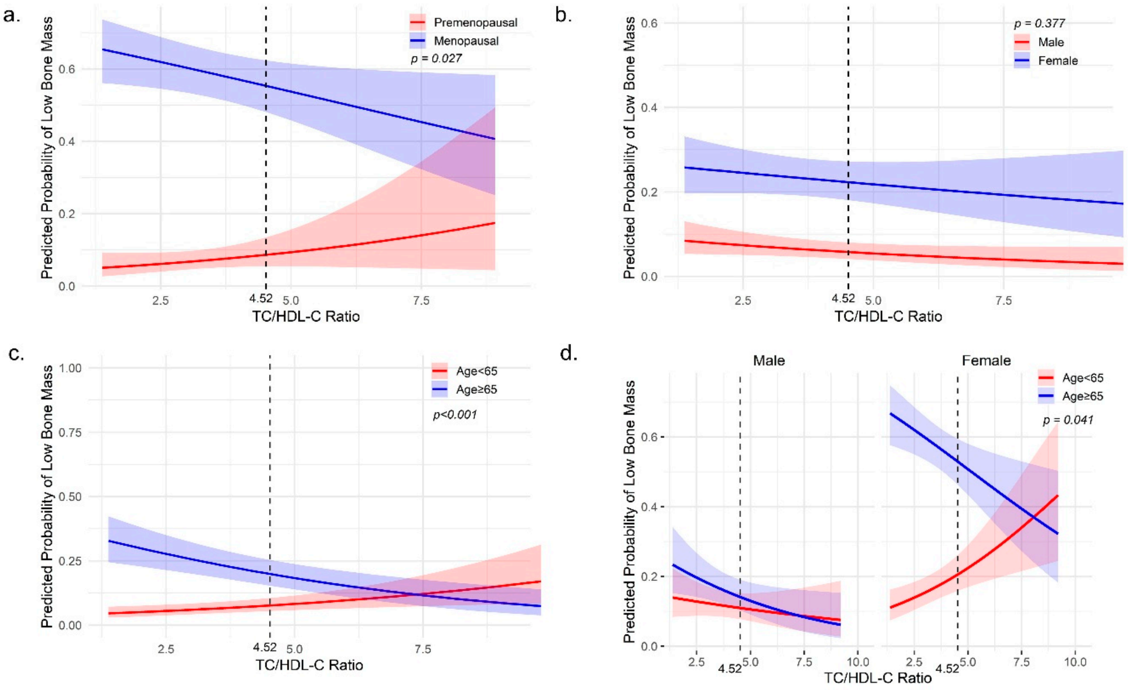

Figure S3. Exploratory interaction analyses of TC/HDL-C ratio with age-defined menopausal status, sex, and age in relation to study-defined total-body T-score-based low-bone-mass phenotype after exclusion of extreme TC/HDL-C observations. (a) Age-defined menopausal status (red: premenopausal; blue: menopausal). (b) Sex (red: male; blue: female). (c) Age (red: age < 65 years; blue: age ≥ 65 years). (d) Predictions by age group and sex from the three-way inter-action model (red: age < 65 years; blue: age ≥ 65 years). The vertical dashed line at TC/HDL-C = 4.52 marks the turning point from the overall outlier sensitivity spline analysis for reference. Solid curves represent model-predicted probabilities, and shaded areas indicate pointwise 95% confidence intervals. The p value in panel D is for the overall three-way interaction. Covariate adjustment and prediction settings are described in Section 2.5. Panel B adjusts for continuous age; panels A, C, and D do not additionally include continuous age. Panel A is restricted to women.

Table S1. Association between lipid profiles and study-defined T-score-based low-bone-mass phenotypes in adults using logistic regression.

Variables, unit	Total-body low bone mass (T-score < -1.0)						Central-site low bone mass (any T-score < -1.0)					
	Model 1[a] OR (95% CI)	*p*	Model 3 [b] OR (95% CI)	*p*	Model 4 [c] OR (95% CI)	*p*	Model 1 [a] OR (95% CI)	*p*	Model 3 [b] OR (95% CI)	*p*	Model 4 [c] OR (95% CI)	*p*
HDL-C, per 10 mg/dL	1.265(1.213,1.320)*	<0.001	1.081(1.018,1.148)*	0.011	1.055(0.998,1.115)	0.060	1.110(1.073,1.147)*	<0.001	1.065(1.016,1.117)*	0.009	1.048(1.003,1.096)*	0.036
TC, per 10 mg/dL	1.027(1.009,1.046)*	0.003	1.003(0.980,1.026)	0.816	1.005(0.983,1.028)	0.647	1.015(1.001,1.029)*	0.036	1.013(0.996, 1.030)	0.141	1.014(0.997,1.031)	0.105
TG, per 10 mg/dL	0.989(0.980,0.998)*	0.019	-	-	1.006(0.995,1.018)	0.273	0.998(0.992,1.004)	0.539	-	-	1.003(0.996,1.011)	0.409
LDL-C, per 10 mg/dL	0.994(0.974,1.013)	0.524	0.985(0.962,1.010)	0.235	0.986(0.963,1.010)	0.263	1.000(0.986,1.015)	0.976	1.004(0.986,1.022)	0.650	1.004(0.987,1.022)	0.632

TC/HDL-C	0.786(0.739,0.836)*	<0.001	0.918(0.835,1.010)	0.078	0.970(0.898,1.047)	0.432	0.916(0.878,0.956)*	<0.001	0.966(0.904,1.032)	0.303	0.989(0.937,1.045)	0.702

Sensitivity analysis was conducted after removing 16 extreme TC/HDL-C observations (±3 IQR). *Statistically significant association ($p < 0.05$). [a] Unadjusted. [b] Adjusted for age, sex, BMI, HbA1c, Glu, TG, UA, Ca, VitD, eGFR, smoking status, education level, monthly average income level, DM, fracture history, hypertension, dyslipidemia, regular use of corticosteroids. [c] Adjusted for covariates included in Model 3 except TG. The total-body phenotype was operationally defined as a total-body BMD T-score < -1.0. The central-site phenotype was operationally defined as a T-score < -1.0 based on the lower of the right or left femoral neck T-scores or the mean lumbar spine (L1–L4) T-score. Abbreviations: OR, odds ratio; CI, confidence interval; *p*, p-value; -: not available. BMI, body mass index; HbA1c, Glycated hemoglobin; Glu, Fasting plasma glucose; TC, Total cholesterol; HDL-C, High-density lipoprotein cholesterol; TC/HDL-C, Total cholesterol to high-density lipoprotein cholesterol ratio; TG, Triglyceride; Ca, Calcium; VitD, 25-hydroxyvitamin D; UA, Uric acid; eGFR, Estimated glomerular filtration rate; DM, diabetes.

Table S2. Exploratory threshold effect analyses of the TC/HDL-C ratio and per-unit LDL-C in relation to the study-defined total-body T-score-based low-bone-mass phenotype after exclusion of extreme TC/HDL-C observations.

	Model 1[a]		Model 2[b]		Model 3[c]	
Population with turning point	OR (95% CI)	*p*	OR (95% CI)	*p*	OR (95% CI)	*p*
All participants						
TC/HDL-C≤4.52	0.739(0.663,0.823)*	<0.001	0.901(0.790,1.027)	0.119	0.813(0.699,0.947)*	0.008
TC/HDL-C>4.52	1.006(0.828,1.221)	0.955	1.226(0.980,1.534)	0.075	1.222(0.948,1.576)	0.122
LDL-C≤126.71	0.995(0.991,0.999)*	0.007	0.995(0.991,1.000)	0.057	0.993 (0.988,0.998)*	0.010
LDL-C >126.71	1.003(0.999,1.008)	0.167	1.002(0.996,1.007)	0.584	1.002 (0.997,1.008)	0.420
Male participants						
TC/HDL-C≤4.36	0.586(0.463,0.742)*	<0.001	0.843(0.654,1.086)	0.187	0.803(0.605,1.066)	0.128
TC/HDL-C>4.36	1.074(0.798,1.445)	0.637	1.158(0.850,1.576)	0.353	1.136(0.792,1.628)	0.488
LDL-C≤117.33	1.000(0.997,1.003)	0.987	0.997(0.993,1.001)	0.163	0.996(0.992,1.001)	0.093
LDL-C>117.33	1.001(0.997,1.004)	0.715	1.000(0.996,1.004)	0.859	0.998(0.993,1.002)	0.275
Female participants						
TC/HDL-C≤4.21	1.002(0.864,1.163)	0.978	0.876(0.728,1.054)	0.161	0.778(0.626,0.967)*	0.023

TC/HDL-C>4.21	1.124(0.908,1.393)	0.283	1.206(0.929,1.567)	0.160	1.267(0.933,1.720)	0.129
LDL-C≤137.86	0.995(0.991,0.999)*	0.019	0.995(0.990,1.000)	0.054	0.991(0.986,0.997)*	0.002
LDL-C>137.86	1.000(0.993,1.007)	0.991	0.997(0.989,1.005)	0.488	0.997(0.988,1.006)	0.468

Sensitivity analysis was conducted after removing 16 extreme TC/HDL-C observations (±3 IQR). *Statistically significant association ($p < 0.05$). [a] Unadjusted. [b] Adjusted for age, sex, and BMI. [c] Adjusted for age, sex, BMI, HbA1c, Glu, TG, UA, Ca, VitD, eGFR, smoking status, education level, monthly average income level, DM, fracture history, hypertension, dyslipidemia, regular use of corticosteroids. Abbreviations: OR, odds ratio; CI, confidence interval; *p*, p-value; BMI, body mass index; HbA1c, Glycated hemoglobin; Glu, Fasting plasma glucose; TC, Total cholesterol; HDL-C, High-density lipoprotein cholesterol; TC/HDL-C, Total cholesterol to high-density lipoprotein cholesterol ratio; TG, Triglyceride; Ca, Calcium; VitD, 25-hydroxyvitamin D; UA, Uric acid; eGFR, Estimated glomerular filtration rate; DM, diabetes.

Table S3. Critical design effects (DEFF_crit) for all reported associations between lipid indicators and the study-defined T-score-based low-bone-mass phenotypes.

**Panel A. Associations between lipid indicators and low-bone-mass phenotypes**							
**Lipid indicator**	**Source**	**Total-body low bone mass**			**Central-site low bone mass**		
		**Model 1**	**Model 3**	**Model 4**	**Model 1**	**Model 3**	**Model 4**
HDL-C, per 10 mg/dL	Table 2	31.23	1.66	NS	9.98	1.69	1.24
	Table S1	30.93	1.68	NS	9.79	1.77	1.12
TC, per 10 mg/dL	Table 2	2.36	NS	NS	1.01	NS	NS
	Table S1	2.19	NS	NS	1.17	NS	NS
TG, per 10 mg/dL	Table 2	1.11	-	NS	NS	-	NS
	Table S1	1.48	-	NS	NS	-	NS
LDL-C, per 10 mg/dL	Table 2	NS	NS	NS	NS	NS	NS
	Table S1	NS	NS	NS	NS	NS	NS
TC/HDL-C ratio	Table 2	13.33	NS	NS	4.42	NS	NS
	Table S1	15.25	NS	NS	4.25	NS	NS
**Panel B. Threshold (segmented) analyses in relation to the total-body phenotype**							

Population	Segment	Source	Model 1	Model 2	Model 3
All participants	TC/HDL-C≤4.39	Table 3	6.08	NS	1.60
	TC/HDL-C≤4.52	Table S2	7.83	NS	1.86
	TC/HDL-C>4.39	Table 3	NS	1.85	1.30
	TC/HDL-C>4.52	Table S2	NS	NS	NS
	LDL-C≤126.62	Table 3	1.55	1.23	1.95
	LDL-C≤126.71	Table S2	1.55	1.23	1.95
	LDL-C >126.62	Table 3	NS	NS	NS
	LDL-C >126.71	Table S2	NS	NS	NS
Male participants	TC/HDL-C≤4.55	Table 3	NS	NS	NS
	TC/HDL-C≤4.36	Table S2	5.14	NS	NS
	TC/HDL-C>4.55	Table 3	NS	NS	NS
	TC/HDL-C>4.36	Table S2	NS	NS	NS
	LDL-C≤119.75	Table 3	NS	NS	NS
	LDL-C≤117.33	Table S2	NS	NS	NS
	LDL-C>119.75	Table 3	NS	NS	1.23

	LDL-C>117.33	Table S2	NS	NS	NS
Female participants	TC/HDL-C≤4.11	Table 3	NS	NS	NS
	TC/HDL-C≤4.21	Table S2	NS	NS	1.33
	TC/HDL-C>4.11	Table 3	NS	1.66	1.23
	TC/HDL-C>4.21	Table S2	NS	NS	NS
	LDL-C≤137.39	Table 3	1.55	NS	2.66
	LDL-C≤137.86	Table S2	1.55	NS	2.66
	LDL-C>137.39	Table 3	NS	NS	NS
	LDL-C>137.86	Table S2	NS	NS	NS

Abbreviations: DEFF_crit, critical design effect, calculation method in methods; TC, total cholesterol; TG, triglycerides; HDL-C, high-density lipoprotein cholesterol; LDL-C, low-density lipoprotein cholesterol. NS: not reach significance under model-based variance estimation; -: not available. Panel A: Model 1, unadjusted; Model 3 and Model 4, progressively adjusted models as specified in Table 2. Source rows correspond to Table 2 (primary analysis) and Supplementary Table S1 (sensitivity analysis). Panel B: turning points were estimated from the data and differ between Table 3 (primary threshold analysis) and Supplementary Table S2 (after exclusion of extreme TC/HDL-C observations).
